# The Effect of Angle and Velocity on Change of Direction Biomechanics: An Angle-Velocity Trade-Off

**DOI:** 10.1007/s40279-018-0968-3

**Published:** 2018-08-09

**Authors:** Thomas Dos’Santos, Christopher Thomas, Paul Comfort, Paul A. Jones

**Affiliations:** 1 Human Performance Laboratory, Directorate of Sport, Exercise, and Physiotherapy, University of Salford, Greater Manchester, UK; 2 School of Health, Sport and Professional Practice, University of South Wales, Pontypridd, Wales UK

## Abstract

Changes of direction (CODs) are key manoeuvres linked to decisive moments in sport and are also key actions associated with lower limb injuries. During sport athletes perform a diverse range of CODs, from various approach velocities and angles, thus the ability to change direction safely and quickly is of great interest. To our knowledge, a comprehensive review examining the influence of angle and velocity on change of direction (COD) biomechanics does not exist. Findings of previous research indicate the biomechanical demands of CODs are ‘angle’ and ‘velocity’ dependent and are both critical factors that affect the technical execution of directional changes, deceleration and reacceleration requirements, knee joint loading, and lower limb muscle activity. Thus, these two factors regulate the progression and regression in COD intensity. Specifically, faster and sharper CODs elevate the relative risk of injury due to the greater associative knee joint loading; however, faster and sharper directional changes are key manoeuvres for successful performance in multidirectional sport, which subsequently creates a ‘performance-injury conflict’ for practitioners and athletes. This conflict, however, may be mediated by an athlete’s physical capacity (i.e. ability to rapidly produce force and neuromuscular control). Furthermore, an ‘angle-velocity trade-off’ exists during CODs, whereby faster approaches compromise the execution of the intended COD; this is influenced by an athlete’s physical capacity. Therefore, practitioners and researchers should acknowledge and understand the implications of angle and velocity on COD biomechanics when: (1) interpreting biomechanical research; (2) coaching COD technique; (3) designing and prescribing COD training and injury reduction programs; (4) conditioning athletes to tolerate the physical demands of directional changes; (5) screening COD technique; and (6) progressing and regressing COD intensity, specifically when working with novice or previously injured athletes rehabilitating from an injury.

## Key Points

**Table:** 

Change of direction angle and approach velocity are critical factors that impact the directional change technical execution, deceleration and reacceleration requirements, knee joint loading, and lower limb muscle activity. Thus, these two factors regulate the progression and regression in change of direction intensity.
An ‘angle-velocity trade-off’ exists during change of direction, whereby faster approaches compromise the execution of the intended directional change.
Change of direction biomechanical demands are ‘angle’ and ‘velocity’ dependent; therefore, practitioners and researchers should understand the implications of these two factors when coaching and screening change of direction technique, creating and implementing strength and conditioning programs, and interpreting change of direction research.

## Introduction

The ability to change direction efficiently is central to the success of multidirectional sports [1–6]; however, changing direction has also been identified as a primary action resulting in non-contact anterior cruciate ligament (ACL) injury [7–14]. Athletes perform a diverse range of change of direction (COD) angles, at a variety of approach velocities in sport [1, 5, 15–18]. A plethora of biomechanical investigations has investigated a spectrum of angled direction changes (30°–180°), at various approach velocities (~3–7 m·s^−1^), in an attempt to provide insight into the biomechanical risk factors associated with increased injury risk and the mechanics required for faster performance (Tables 1, 2, 3, 4). However, it worth noting that COD angle and approach velocity are critical factors influencing COD biomechanics, and include knee joint loading, whole body kinetics and kinematics, ground reaction force (GRF) characteristics, muscle activation, velocity of centre of mass, deceleration and propulsive requirements, technical, and task execution of the COD (Tables 1, 4). This should be acknowledged when interpreting the biomechanical literature.

**Table 1:** Summary of studies that have investigated the effect of angle on COD biomechanics

Study	Subjects	COD tasks and angle comparisons	Effect of increased COD angle
Pre-planned			
Havens and Sigward [25]	25 healthy soccer players	45° cut after 7.5 m (CUT45)	↑ FFC GCT in CUT90 (*p* < 0.001, *d* = *2.17*)
		90° cut after 7.5 m (CUT90)	Braking
		PP, As fast as possible	↑ Posterior GRI CUT90 (*p* < 0.001, *d* = 2.42)
			↑ PFC vs. FFC impulse for CUT90 (*p* < 0.001)
			↑ GRF in FFC and PFC during CUT90 (*p* < 0.001, *d* = 0.97–2.39)
			↑ PFC vs. FFC GRF for CUT90 (*p* < 0.001)
			Translation phase
			↑ FFC GRI in CUT90 (*p* < 0.001) but ↓ during PFC (*p* < 0.001).
			↓ GRI in PFC for both cuts (*p* < 0.001)
			↑ ML COM-COP separation distance in CUT90 (*p* < 0.001, *d* = 2.49).
Havens and Sigward [30]	25 healthy soccer players (12 females)	45° and 90° cut—PP, As fast as possible	↑ KAMs (−1.83 ± 0.77 vs. −1.07 ± 0.99 N.m.kg^−1^, *d* = 0.86)
Havens and Sigward [26]	25 healthy soccer players	45° cut after 7.5 m (CUT45)	↔ differences between sexes
		90° cut after 7.5 m (CUT90)	Deceleration: sagittal plane mechanics (*p* < 0.001)
		PP, As fast as possible	↓ approach velocity (*d* = −4.05)
			↓ hip and knee flexion (*p* < 0.001) at IC (*d* = −1.52 to 1.99)
			↑ ankle plantar flexion angles at IC (*d* = 0.80)
			↓ Hip sagittal excursion (*d *= −1.68), ↑ knee and ankle excursion (*d* = 1.03–1.90)
			↓ hip extensor (*d* = 1.22) and ankle plantar flexor moments (*d* = 0.83)
			↓ ankle power absorption (*d *= −1.04), ↑ knee power absorption in sag plane (*d* = 0.99)
			Redirection: frontal and transverse plane mechanics (*p* < 0.001)
			↑ hip abduction (*d* = 3.31)
			↑ trunk lean angles at IC (*d* = 1.02)
			↑ hip adductor moment (*d* = 1.32)
			↔ diff in hip frontal power
			↑ pelvic rotation (48.7 ± 2.4° vs. 14.2 ± 5.5° *p* < 0.001, *d* = 8.13)
Besier et al. [28]	11 healthy male	Sidestepping 30° and 60	↑ KAMs^a^
		~3 m·s^−1^, PP and UP	
McLean et al. [20]	10 male and female	Jump landing	↑ KVA, Hip and knee flexion
	College level	45°, 180° shuttle	
		4.5–5.5 m·s^−1^—PP	
Hader et al. [24]	Twelve highly-trained young soccer players	45° and 90° cut—PP	Speed-related variables -angle-dependent with likely ↓ peak speed, almost certainly ↓ speed during COD and ↑ completion time
			Minimum speed reached during COD was a large-to-very-large determinant of CUT45 and CUT90 peak acceleration and peak speed additionally contributed to CUT45 and CUT90.
Schot et al. [19]	12 6 men and women	45 and 90° cuts—PP	↑ average braking forces −39% (*p* < 0.001, *d* = 1.72)
			↑ average propelling forces −56% (*p* < 0.001, *d* = 2.13)
			↓ vertical forces (*p* < 0.001. *d* = 1.33)
Hader et al. [23]	12 soccer players	45° or 90° cut after 10 m—PP	↓ metabolic demand (estimate energy expenditure)
			↑ muscle activity for VL and BF
			↑ deceleration distances (7.1 ± 1.2 m vs. 4.3 ± 1.9 m, *d* = *1.76)*
Schreurs et al. [22]	13 males and 16 females	45°, 90°, 135° and 180° PP	Both sexes—knee flexion moment ↓ (*p* < 0.05, ES = 0.04)
		As fast as possible	Both sexes—↑ knee valgus moment (*p* < 0.01, ES = 0.37) and then stabilized (45° vs. ≥ 90°)
		Male average speed 4.7, 3.8, 3.5 and 3.4 m·s^−1^, females average velocity 4.2, 3.6, 3.3 and 3.2 m·s^−1^	Both sexes—↓ vGRF (*p* < 0.01, ES = 0.11).
			Males ↔ knee flexion
			Females -↓ knee flexion (*p* < 0.01, ES = 0.07)
			Both sexes—↑ completion time increased when cutting angle increased (both *p* < 0.01, ES = 0.87 and ES = 0.94)
			Both sexes—↓ approach velocity
Unplanned			
Cortes et al. [29]	Nineteen female collegiate soccer athletes	45° SS, 180° pivot and drop landing—UP	↓ knee flexion (*d* = −2.02), ↑ KVA (at max VGRF) (*d* = 0.47), ↑ PPGRF (*d* = 2.35), ↑ internal varus moments at peak stance (*p* < 0.001, *d* = 0.74)
		Min 3.5 m·s^−1^	
Sigward, Cesar and Havens [27]	Forty-five soccer athletes (20 females)	45° and 110° cuts	↑ KVMs (*p* < 0.001)—2.4 times greater^b^
		4.5–5.5 m·s^−1^—UP	↑ GRF (21% vertical, 87% posterior and 227% lateral greater) *(p* < 0.001)^b^
			↑ Hip abduction angle (*p* < 0.001) ↓ Hip internal rotation *(p* = 0.03)^b^
			↑ pelvic rotation (37.15 ± 3.32 vs. 6.95 ± 1.20°)^b^

*SS* sidestep, *COM* centre of mass, *KVM* knee valgus moment, *KAM* knee abduction moment, *GCT* ground contact time, *GRF* ground reaction force, *PPGRF* peak posterior GRF, *PFC* penultimate foot contact, *FFC* final foot contact, *GRI* ground reaction force impulse, *COM*-*COP* centre of mass–centre of pressure, *KVA* knee valgus angle, *PP* pre-planned, *UP* unplanned, *IC* initial contact, *COD* change of direction, *VL* vastus lateralis, *BF* biceps femoris

^a^Raw values not provided

^b^Effect size cannot be calculated as SD not provided

**Table 2:** Plant phase ground contact times in different angled COD tasks

Study	Subjects	COD Task	Ground contact time (s)	Training recommendations
Green et al. [111]	Male rugby union	45° cut—PP	0.213 ± 0.03 to 0.241 ± 0.03	Fast SSC/ Fast reactive strength (< 0.250 s) 1. Drop jumps 2. Bounds 3. Stiff ankle hops 4. Hurdle jumps
Vanrenterghem et al. [31]	Active female	45° cut (actual 39.5–25.5°)—PP (2, 3, 4 and 5 m·s^−1^)	0.45 ± 0.10, 0.28 ± 0.04, 0.24 ± 0.03 and 0.20 ± 0.03	
Havens & Sigward [25]	Male/Female soccer	45° cut (5.83± 0.45 m·s^−1^)	0.157 ± 0.019	
Spiteri et al. [113]	Female basketball	45° cut (reactive, human)	0.23–0.26	Based on training recommendations [83, 111, 148]
Spiteri et al. [106]	Female basketball	45° cut (video)	0.42 ± 0.04 to 0.51 ± 0.05	Unilateral multiplanar plyometrics 1. Angled hops 2. Zig zag bounds 3. Ice skaters 4. Reactive side to side jumps
Kimura & Sakurai [34]	Male basketball	60° cuts—slow vs. fast velocity—PP	Fast: 0.206 ± 0.021	
		(4.49 ± 0.42 and 5.83 ± 0.32 m·s^−1^)	Slow: 0.235 ± 0.017	
Condello et al. [52]	Male/Female soccer	60° cut—PP	Male 0.233 ± 0.03	Based on training recommendations [140]
			Female 0.237 ± 0.03	
Kristianslund et al. [32]	Female handball	67±14˚ (sports specific cut with ball)—PP	0.319 ± 0.062	Combination of fast and slow reactive strength exercises—bordering fast and slow SSC classification [148]
Marshall et al. [40]	Male hurling	75° cut—PP	0.371 ± 0.059	
Maloney et al. [112]	Male recreationally active	90° cut—PP	Faster: 0.25 ± 0.04	
		Faster vs. slower comparisons	Slower: 0.31 ± 0.05	
Havens & Sigward [25]	Male/Female soccer	90° cut—PP	0.252 ± 0.059	
Spiteri et al. [106]	Female basketball	90° cut into shuffle—PP	0.32 ± 0.03 to 0.35 ± 0.03	
Jones et al. [36]	Female soccer	90° cut—PP (4.40 ± 0.22 m·s^−1^)	0.261 ± 0.045	
Nedergaard et al. [35]	Male soccer	135° v cut—PP (3.82–4.82 m·s^−1^)	0.388 ± 0.072 to 0.496 ± 0.115	Slow SSC actions, slow reactive strength (>0.250 s): ballistic exercises, weightlifting derivatives 1. Countermovement jumps 2. Broad Jumps 3. Jump squats 4. Weightlifting derivatives including: jump shrugs and hang clean pulls Based on training recommendations [81, 83, 98, 101, 108, 148]
Sasaki et al. [42]	Male soccer	180° turn—PP	0.44 ± 0.07	
Spiteri et al. [106]	Female basketball	180° turn—PP	0.42 ± 0.03 to 0.47 ± 0.04	
Jones et al. [36]	Female soccer	180° turn—PP (4.03 ± 0.2 m·s^−1^)	0.517 ± 0.082	
Dos’Santos et al. [59]	Male team sport	180° turn—PP	0.46 ± 0.10	

Note: Strength training should not be omitted and overlooked, as an underpinning foundation of strength is required for effective use of plyometrics, ballistic training, and weightlifting exercises [81, 83, 98, 101, 108, 148]. Shorter GCTs, and greater braking and propulsive forces have been identified as determinants of faster COD speed performance [40, 42, 59, 83, 106]. In light of these determinants, practitioners should develop their athlete’s ability to express high forces quickly (rate of force development) for faster COD speed performance [41, 81, 100, 105, 107]

*COD* change of direction, *SSC* stretch shortening cycle, *GCT* ground contact time, *PP* pre-planned

**Table 3:** Summary of studies that have examined executed cutting angle

Study	Velocity (m·s^−1^)	COD task—intended angle of COD task	Method of determining cutting angle	Actual angle of COD
Besier et al. [28]	~3	60° cut (SS)—PP	= tan^−1^[(*y*_*i*_ − *y*_*i* −1_)/ [(*x*_*i*_ − *x*_*i* −1_)], where *i* = *i*th time point	56.4° ± 4.4°
			*X* and *y* displacements of the pelvic center (anterior/posterior and medio/lateral disablements)	
Vanrenterghem et al. [31]	2.0, 3.0, 4.0 and 5.0	45° cut (SS)—PP	Angle of COM	34.91°, 29.41°, 23.81° and 17.51°—with increased approach velocities
Condello et al. [52]	As fast as possible	60° cut (SS) (inside angle 120°)—PP	Computed from two-line vectors connecting pelvis centre (midpoint of ASIS) positions projected to the floor (x-y-plane)	~150° inside angle
			Line 1 = 1.5 m before initial plate contact and initial plate contact. Line 2 = Plate push-off and 1.5 m after plate push-off	
Suzuki et al. [55]	As fast as possible	90° SS and XOC—PP	Angle between horizontal velocity vectors of the whole-body COM at foot strike and toe-off	SS = 40.5° ± 8.7°
	3.82 ± 0.28 and 3.67 ± 0.31^a^			XOC = 33.0° ± 6.8°
David et al. [116]	As fast as possible	90° cut (SS)—PP	COM position at touch down and toe off	75.6°
Rovan et al. [66]	2.77	Jog: 30°, 60°, 90°, 120°, 150° and 180°	Difference in direction of COM movement between steps (based on GNSS and data)	Jog: 7.5°, 10.7°,15.0°, 16.2°, 9.6°, 1.5°
	4.16	Running: 30°, 60°, 90°, 120°, 150° and 180°—PP		Running: 6.9°, 12.7°, 14.6°, 7.0°, 8.3°, 3.2°

*COD* change of direction, *SS* sidestep, *XOC* crossover cut, *COM* centre of mass, *ASIS* anterior superior iliac spine, *GNSS* global navigation satellite system, *PP* pre-planned

^a^Velocity at foot strike

**Table 4:** Summary of studies that have investigated the effect of velocity on COD biomechanics

Study	Subjects	COD task	Velocity (m·s^−1^) comparisons	Method of determining approach velocity	Effect of faster approach velocity
Vanrenterghem et al. [31]	Fourteen female participants	45° SS–PP	2.0, 3.0, 4.0 and 5.0	Timing cells placed 2 m apart and 2 m away from the cut—and Velocity of COM	COM travel angle achieved more poorly −34.9°, 29.4°, 23.8° and 17.5° approach speeds of: 2, 3, 4 and 5 m·s^−1^
					↑ KVMs (*p* < 0.05)
					↑ Knee flexion angle (different at 5 m·s^−1^), ↓ GCT, ↑ Peak posterior and Medial GRF (*p* < 0.0005)
Dai et al. [33]	Thirty-six recreational athletes	45° SS–PP	3.80 ± 0.35 and 2.10 ± 0.33	Speed of centre of pelvis (middle of right and left ASIS and PSIS)	↓ GCT and knee flexion ROM (*p* < 0.001),
					↑ PPGRF, knee extension moment at PPGRF, knee valgus angle and varus moment at PPGRF, knee joint stiffness and peak knee flexion angle (*p* ≤ 0.001)
Kimura and Sakurai [34]	Seven male university basketball	60° SS–PP	5.83 ± 0.32 and 4.49 ± 0.42	Timing cells placed 2 m apart and 2 m away from the cut and speed of whole body COM	↑ posterior PFC impulse, peak external flexion moment, ↑ greater knee flexion in PFC (*p* < 0.05)
					↑ KVM (*p* < 0.05)
					↓ GCTs in PFC and FFC (*p* < 0.05)
Nedergaard et al. [35]	10 male soccer players	135° v cut—PP	3.82 ± 0.36, 3.97 ± 0.39, 4.39 ± 0.48, 4.40 ± 0.77, 4.82 ± 0.58	Timing gates 3 m apart—0.5 m prior to COD	↑ KVM
					↑ trunk decelerations and during all three-foot contacts (*p* ≤ 0.05)
					↑ peak ankle and knee velocities across steps (*p* ≤ 0.05)
					↔ in PPGRF or peak extensor moments
Kristianslund et al. [32]	123 female handball players	67 ±14° SS past a defender (with a ball)—PP		Absolute speed of COM at IC	Regression analysis—approach speed factor associated with grater KAMs
					An increase in approach speed of 1 SD increased the knee abduction moment ~13%

*SS* sidestep, *COM* centre of mass, *KVM* peak knee valgus moment, *GCT* ground contact time, *GRF* ground reaction force, *PPGRF* peak posterior GRF, *PFC* penultimate foot contact, *FFC* final foot contact, *KAM* peak knee abduction moments, *COD* change of direction, *IC* initial contact, *SD* standard deviation, *PP* pre-planned

The biomechanical demands of changes of directions (CODs) are described as ‘angle dependent’ and ‘velocity dependent’, whereby the technical execution and whole body kinetics and kinematics are likely to differ between different angled CODs [19–30], and also influenced by the approach velocity [31–35]. Thus, the purpose of this review was to examine the effect of angle and velocity of CODs on various biomechanical parameters including GRF properties, joint kinetics and kinematics, performance (time), injury risk factors (knee abduction moments, knee abduction angle), task execution (executed angle of COD), and muscle activation. A further aim was to discuss the implications of these factors on coaching COD technique, strength and conditioning training prescription, screening COD technique, and progression/regression of COD intensity when prescribing COD training. In addition, this review discusses the concept of an ‘angle-velocity trade-off’ when changing direction. Understanding the mechanics associated with faster performance and injury risk reduction are of great interest to practitioners, thus highlighting the importance of this review. For the purpose of this review, a sidestep involves a lateral foot-plant opposite to the direction of travel. Conversely, a crossover cut involves using the plant foot corresponding towards the same direction of travel. Finally, a pivot is a bilateral turning strategy where one foot rotates and remains in contact with the ground (typically for directional changes ≥ 135°).

## Effect of Angle on COD Biomechanics

Numerous directional changes of various angles are performed in sport [1, 15–17] and, as such, have been extensively examined to gain an understanding of the associated injury risk factors [27, 32, 36–39] and kinematic and kinetic determinants of faster performance [30, 40–42]. Forty-five-degree sidesteps have been thoroughly examined across the literature [22, 25–27, 31, 39, 41, 43–48], with directional changes of 30° [28, 49–51], 60° [34, 50, 52, 53], 67° [32], 75° [40], 90° [22, 25, 26, 36, 38, 54, 55], 110° [27, 48, 56], 135° [22, 35] and 180° [22, 29, 36, 37, 47, 57–60] also being investigated. However, the biomechanical demands of CODs are angle dependent (Table 1), as the angle of COD influences the magnitude of knee joint loading [22, 27–30], affects the deceleration and reacceleration requirements of the COD [23–26, 30], influences the magnitude of braking and propulsive forces [19, 22, 25, 27, 29], and impacts the orientation of the force vector to perform the COD [25]. Moreover, the angle of the COD results in different techniques [26, 30] and joint and segmental differences in order to execute the directional change [26], while also influencing lower limb muscle activity and estimated energy expenditure [23].

### Ground Reaction Force Characteristics and Whole-Body Centre of Mass Velocity are Angle Dependent

Researchers have shown that the COD angle influences the braking and propulsive force characteristics of the final foot contact (FFC) (plant phase) [19, 22, 25, 27, 29] and also the braking force characteristics of the penultimate foot contact (PFC) (step prior to plant phase) [25] (Table 1). Schot et al. [19] reported significantly greater average braking forces and propulsion forces during a 90° cut compared to a 45° cut. Likewise, vertical, posterior and lateral GRFs were 21%, 87% and 228% greater, respectively, during a 110° cut in comparison to a 45° cut in both male and female soccer players [27]. These results not only confirm that the GRF magnitudes are significantly greater with sharper cuts, but the direction requirements of the force are different (i.e. greater posterior and laterally directed force for sharper CODs). Conversely, Schreurs et al. [22] documented significantly (*p* < 0.01) greater vertical GRF in 45° cuts in comparison to sharper CODs (90°, 135° and 180°). However, GRF is a three-component vector, but a downfall of the work by Schreurs et al. [22] is only the vertical component of GRF was examined.

**Fig. 1:**
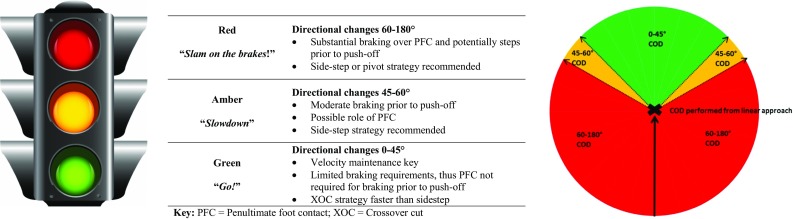
Traffic light system indicating braking strategy and technique requirements for different angled directional changes based on a linear approach. Based on the results of previous research [23–25, 34–38, 55, 58–61, 66]

To our knowledge, only one study has directly compared the GRF characteristics of the PFC between different angled CODs [25], observing greater posterior braking GRFs and ground contact times (GCTs), thus ground reaction impulse (GRI) during the 90° cut in both PFC and FFC compared to 45° cutting. Interestingly, posterior GRF and impulse were greater in the PFC compared to the FFC during the 90° cut; however, this result was not the case for the 45° cut, whereby the braking forces were more evenly distributed across both foot contacts. These findings support Andrews et al. [61] description of cutting as a multi-step action and highlight the importance of the braking forces during the PFC for sharper cuts. Researchers have reported that greater braking force characteristics over the PFC were associated with lower knee joint loads in the cutting or turning limb during 90° cuts and 180° turns [36]. Faster 180° performance has also been associated with greater PFC horizontal braking forces [58–60], while substantial braking forces have also been reported in the PFC during 60° [34] and 135° [35] CODs. Collectively, the braking force characteristics of CODs are ‘angle dependent’, with a limited role of the PFC when changing direction ≤ 45°, but a prominent role for CODs ≥ 60° during pre-planned tasks (Fig. 1). Unfortunately, however, the results of Jones et al. [62] indicate that unanticipated situations do not allow postural adjustments prior to the FFC to evoke greater braking force characteristics during the PFC; however, it should be noted that the unanticipated COD task involved responding to a light stimuli, which is more challenging than using a sports-specific stimulus [63, 64], and it also lacks specificity to the sporting situations where athletes typically scan and process kinematic and postural cues prior to changing direction [65]. Further research is warranted investigating the role of the PFC during unanticipated tasks utilising sports-specific stimuli.

Havens and Sigward [25] also examined the kinetic profile over the translation phase (shift from sagittal to frontal plane) and reported greater medio-lateral (ML) GRFs and longer GCTs and, thus, greater ML GRI demonstrated during the 90° cut. Normative GCTs are presented in Table 2 for different angled directional changes indicating a longer GCT with increased angle. The longer GCTs could be attributed to sharper CODs requiring longer braking force application, therefore braking impulse (impulse = force × time, thus change in momentum), in order to reduce the velocity (i.e. change in momentum) and redirect the athlete into the new intended direction [25, 26]. Interestingly, the greater ML GRI observed by Havens and Sigward [25] was accompanied with greater ML centre of mass–centre of pressure (COM-COP) distances, suggesting athletes modify their COM-COP distances via greater lateral foot-plant distances and trunk lean into the intended direction to generate ML force and impulse (Table 1). This observation is supported by previous research reporting a strong relationship (*r* = 0.59) between lateral foot-plant distance and peak medial GRF during 90° cutting [38]. Consequently, these results suggest individuals modify their stance time and GRFs, thus impulse (during deceleration and reacceleration phases), and modify their COM-COP differently, and, accordingly, to the angle of the COD.

### Whole-Body Centre of Mass Velocity and Deceleration Requirements are Angle Dependent

The angle of COD can also impact the velocity profile when changing direction (i.e. approach velocity and exit velocity), whereby sharper CODs result in reduced approach velocities and exit velocities [22, 24–26] (Table 1 and Fig. 1). Havens and Sigward [25] reported lower velocities at initial contact (IC) of the PFC and FFC during a 90° cut compared to a 45° cut. Similarly, Hader et al. [24] also observed lower approach velocities and lower velocities during the COD when comparing 90° and 45° cuts. Furthermore, Hader et al. [23] also reported longer deceleration distances for 90° cuts compared to 45° cuts (Table 1). These results support previous studies that have highlighted the importance of preliminary deceleration prior to sharper CODs [35, 61, 66]. The lower approach velocities observed during sharper CODs [22, 24–26] are most likely explained by greater braking forces in the PFC and FFC to reduce the velocity (i.e. change in momentum) and execute the intended COD. Therefore, COD angle will largely determine the velocity that can be maintained and govern the subsequent deceleration requirements of the directional change [23–26] (Fig. 1).

### Effect of COD Angle on Joint Kinetics and Kinematics

When changing direction, the technique chosen is also angle dependent (Table 1); however, a limited number of studies have compared whole-body kinematics and kinetics between different angled CODs [26]. Havens and Sigward [26] reported joint and segmental differences during the deceleration and reacceleration phases of 45° and 90° cuts (Table 1). Notably, the authors’ results demonstrated that the deceleration demands of a 90° cut may not be evenly distributed across all joints, with a greater reliance on the knee. This finding is concerning because greater peak knee extensor moments, peak posterior GRF and increased quadriceps activity can increase anterior tibial shear force [67], thus ACL loading [68–72]. However, biomechanical deficits in the sagittal plane alone cannot rupture the ACL [73], but a combination of loading in several planes is required [74–77].

Interestingly, during the redirection phase greater hip abduction, greater trunk lean angles at IC and greater hip adductor moments were observed during the 90° cut [26]. Greater hip abduction has been reported to be a biomechanical risk factor associated with knee abduction moments (KAM—synonymous with knee valgus moments for the purpose of this review) [39], thus ACL injury risk [78], and is also a commonly observed visual characteristic of non-contact ACL injuries [9, 79]. This abducted lower extremity position is often discouraged in ACL injury prevention programmes [44], but increased hip abduction is necessary to create a larger ML COM-COP (to create a lateral foot plant) distance [26, 27] and subsequent lateral propulsion for executing sharper CODs [25, 38, 80]. This may create a ‘performance-injury conflict’ from a technique perspective, whereby a greater hip abducted position is necessary to create greater ML COM-COP distances and generate ML forces, but concurrently elevates injury risk due to the potential to generate larger KAMs, because the force vector acts more laterally relative to the knee joint centre [30, 38, 39]. However, this conflict in technique is mediated by an athlete’s physical capacity (i.e. ability to rapidly produce force and neuromuscular control) [58, 81–84] such that stronger athletes with optimal mechanics (i.e. sufficient trunk control, no knee valgus) [32, 37, 38, 44, 45] are able to tolerate the higher loads experienced and could, thus, adopt such techniques.

Havens and Sigward [26] also found hip function to differ between tasks (Table 1). For example, the hip may act as a stabiliser during the 90° cut due to the greater hip adductor moments and relatively low hip frontal and transverse power generation. Conversely, the hip may contribute to propulsion during 45° cuts due to greater hip sagittal and transverse power generation, and transition from hip adductor to hip abductor moments during the stance. Notably, greater pelvic rotation was demonstrated during the 90° cut, approximately 35° more into the new intended direction compared to the 45° [26], similar to research that compared 110° cuts to 45° cuts [27]. These findings suggest that athletes may achieve the greater redirection requirements by rotating their whole body and not just solely the lower limb. From a performance perspective, it may be worthwhile to coach techniques that emphasise whole body rotation and trunk lean towards the intended direction during sharper angled cuts [26, 27, 40], because Marshall et al. [40] reported greater lateral turn (thorax) towards the intended direction was strongly associated with faster 75° COD performance (*r* = 0.51, *p* < 0.01). However, decelerating in the rotated position would result in force absorption and loading in the transverse and frontal planes, in contrast to absorbing and decelerating the force through the sagittal plane, which may be a safer technique [37, 73].

### Sharper CODs Increase the Relative Lower Body Loading

Although the mechanisms of ACL injury are multifactorial [85], COD lower limb and whole-body postures are critical factors associated with knee joint loading [8, 14, 86–89]. Consequently, several investigations have examined the effect of COD angle on associative biomechanical risk factors connected to increased risk of injury (Table 1). Cortes et al. [29] observed greater knee valgus angles, greater peak posterior GRFs, greater internal varus moments, and lower knee flexion angles during a 180° pivot compared to a 45° cut. Likewise, McLean et al. [20] also reported greater knee valgus angles during a 180° pivot compared to a 45° cut. Additionally, Schreurs et al. [22] documented a reduction in knee flexion angle with sharper CODs, and Havens and Sigward [26] reported the knee was primarily involved in absorption during the deceleration phase of sharper cuts. These findings are concerning because extended knee positions can increase anterior tibial shear forces [67], thus increasing ACL strain [68–72]. Moreover, greater knee valgus angles [27, 32, 37, 38, 90] and posterior GRFs [27, 36] are linked to increased KAMs, which can increase ACL strain [68, 91–93]. Collectively, these findings are problematic because KAMs have been shown to prospectively predict non-contact ACL injury in female adolescent athletes [78]. Furthermore, extended knee postures, greater knee abduction angles and greater GRFs have been identified as mechanisms and characteristics linked to ACL injuries [7, 8, 10, 14, 94].

Sigward, Cesar and Havens [27] reported increases in KAMs and GRFs during a 110° cut versus a 45° cut, and differences in hip abduction and internal rotation angle (Table 1). Specifically, KAMs on average were 2.4 times greater during the 110° cut. This finding is corroborated by Besier et al. [28], who reported greater knee valgus loading when comparing 60° sidesteps to 30° sidesteps, and corroborated by Havens and Sigward [30], who demonstrated greater KAMs during 90° cuts compared to 45° cuts (Table 1). The increased KAMs and knee joint loads could be explained by the fact the KAMs are influenced by the magnitude of the GRF, and the moment arm in the frontal plane [32]. Greater GRF magnitudes [19, 25, 27, 29] and lower knee flexion angles [22, 27, 29] have been observed with sharper CODs, yet greater moment arms could be created due to greater hip abduction [26, 27] and greater ML COM-COP distances (i.e. lateral foot plant), which have been reported in sharper CODs [25]. This can have the effect of moving the force vector laterally to the knee, thus creating a larger moment arm relative to the knee joint centre [30, 38, 39]. Consequently, this suggests sharper CODs predispose athletes to greater knee joint loading and subsequent risk of injury, but sharp CODs are unavoidable in sport, and typically performed to evade or pursue opponents or a ball, particularly in unanticipated environments. Thus, it is imperative that athletes have the physical capacity to tolerate the knee joint loading associated with sharper directional changes [58, 81–84] and perform these actions with optimal mechanics [32, 37, 38, 44, 45].

Substantiating the findings of previous studies [27–30], Schreuers et al. [22] demonstrated sharper CODs (90°, 135° and 180°) compared to 45° cuts resulted in greater KAMs in both male and female athletes. However, a noteworthy observation was the stabilisation and lack of differences in KAMs between 90°, 135° and 180° CODs (Table 1), indicating that these tasks may have a similar risk of injury. The authors hypothesised the differences could be attributed to differences in the preliminary steps and knee and trunk positioning relative to the direction of travel. A shortcoming of this study, however, was the authors failed to examine trunk kinematics and did not examine the braking force characteristics of the PFC. Previous studies have shown that the PFC plays an integral role in deceleration prior to executing sharp CODs, such as 60°–90° cuts and 135°–180° turns [25, 34–38, 58–61], and athletes tend to lean and rotate their trunk towards the direction of travel when changing direction [26, 40]. As such, further research comprehensively examining whole-body kinetics and kinematics during the FFC and PFC between different angled CODs is warranted.

Athletes unable to recognise and identify kinematic cues from their opposition or passage of play sooner (lacking perceptual-cognitive ability) [43, 63, 83, 95, 96], in conjunction with sub-optimal physical capacity [58, 81–84] to execute the directional change, may be placed at greater risk of injury, especially during sharper directional changes. However, an athlete who can identify cues earlier could be able to make whole-body postural adjustments for the upcoming movement and pre-activate the required lower-limb and trunk musculature to efficiently execute the direction change, while potentially reducing knee joint loading and subsequent risk of injury [62, 97]. Further research is needed to confirm whether perceptual training (vision training) can enhance deceleration strategies (steps prior to COD) and postural adjustments to facilitate more effective deceleration and safer mechanics to reduce risk of injury and improve performance.

It should be noted that the studies that have investigated the effect of angle on COD biomechanics (Table 1) have all been performed in laboratory settings, with the exception of Hader et al. [23, 24], thus the tasks may not truly reflect competitive situations in multidirectional sport. Nonetheless, when prescribing COD training, athletes should perform shallow CODs before progressing to sharper CODs, due to the elevated knee joint loading and GRFs (Table 1). This is strongly recommended when working with novice or weak athletes (one repetition maximum back squat ≤ 1.5 × body mass), who have limited experience with structured COD training, or may not have the strength capacity to efficiently absorb and tolerate the greater forces and loading associated with sharper CODs [81, 98–101]. Additionally, a progression from shallow to sharper CODs is also advocated for athletes rehabilitating and returning to sport from previous injury.

### Effect on Muscle Activation and Estimated Energy Expenditure

Research from Hader et al. [23] has shown that estimated energy expenditure (EEE) and muscle activity demands of CODs are angle dependent (Table 1). The authors found that as COD angle increased from a straight run to 45° and 90°, the EEE decreased. Furthermore, greater muscle activity (EMG amplitude) of the vastus lateralis and biceps femoris during the sharper cut was also observed. The quadriceps are considered essential for the eccentric contractions during the deceleration phase [23, 61, 102], where GRFs are typically higher during sharper CODs [19, 25, 27, 29]. In addition, hamstring activity is required to stabilise the knee and prevent anterior translation of the tibia, thus protecting the ACL [51, 82, 103, 104]. Collectively, co-contraction of the knee flexors and extensors is required to tolerate the large external loads at the knee when changing direction [51]. As such, due to the greater muscle activations of the knee flexors and extensors during sharper CODs, practitioners should aim to develop knee flexor and extensor strength, in particular eccentric strength [41, 58, 81, 98, 105–109], in athletic populations where CODs are fundamental movements. This may assist and facilitate a greater capacity to absorb the high forces [19, 25, 27, 29] and tolerate the greater knee joint loading [22, 27–30] associated during the deceleration phases of CODs. Furthermore, improvements in strength capacity may improve an athlete’s ability to produce greater braking and propulsive forces and impulse [41, 81, 107, 110], which are determinants of faster performance [41, 59, 60, 106], thus positively enhancing COD performance.

### Effect of COD Angle on Ground Contact Time

Differing from the PFC, the plant step (FFC) contains both a braking and a propulsive force component within the force-time curve [36, 83]. COD angle influences the braking and propulsive forces of the plant step [19, 25, 27, 29] (Table 1); however, COD angle also directly influences GCT [25, 31, 32, 34–36, 40–42, 52, 59, 106, 111–113], thus impacting braking and propulsive impulse during the COD. Table 2 presents normative GCTs reported across the biomechanical literature, illustrating an increased GCT with sharper CODs, which could be explained by the greater braking and propulsive force and impulse requirements to change momentum [23–26]. Furthermore, Condello et al. [52] observed a strong relationship between GCT and executed cutting angle (*r* = 0.60–0.79, *p* < 0.05). Subsequently, this has large implications for the design of strength and conditioning programs and exercise selection for the enhancement of COD performance, which will be discussed in Sect. 6. It should also be noted that approach velocity also affects GCT; this is discussed in Sect. 3.

### COD Task Execution: Intended Angle does not Reflect Executed Angle

Numerous biomechanical investigations select and investigate specific angled direction changes thought to be a key mechanism of ACL injuries or pivotal movements in that sport [25, 31, 32, 34–36, 40–42, 52, 59, 106, 111–113] in an attempt to improve our understanding of the mechanics that influence injury risk and performance. However, although the athletes in these investigations are instructed to perform a directional change of a specific angle (theoretical path), with lines marked on the floor to guide the athlete, the path of travel may not reflect the actual executed angle of COD [28, 31, 52, 55, 114, 115] (Table 3). For example, Vanrenterghem et al. [31] demonstrated COM travel was not achieved during a sidestep, and this was exacerbated with increased running velocities, suggesting there may be an ‘angle-velocity trade-off’ when executing CODs (Table 3) [19]. These findings corroborate previous studies that have also demonstrated the executed COD angle is smaller than the intended COD angle [52, 55, 66, 116] (Table 3). Additionally, Condello et al. [52] inspected the real path of travel during 60° cuts and found athletes performed a rounded or sharp execution of the COD, observing a large correlation (*r* = 0.60–0.79, *p* < 0.05) between performance cutting angle and GCTs; this highlighted that sharper CODs were attributed to longer GCTs, therefore increasing braking and propulsive impulse potential.

Collectively, based on the abovementioned findings (Table 3), in order to achieve the intended direction of travel, the following foot contacts after the plant foot contact (which initiates the COD) must be involved in redirecting the athlete [53, 61, 66, 117]. This concept is substantiated by Rovan et al. [66], who found the following step and two steps after the plant foot contact were involved in facilitating 30°, 60°, 90°, 120°, 150° and 180° CODs based on global positioning system data and qualitative analysis using high speed cameras. Interestingly, the directional change was initiated one step before the plant foot contact, highlighting the role of the PFC for pre-planned CODs. Consequently, the results of Rovan et al. [66] highlight the steps following and prior to the plant foot, and also play an integral role when changing direction, therefore confirming Andrews et al. [61] ’s early description of CODs as a multi-step action. Current COD technique and coaching guidelines typically focus on the plant step (FFC) [3, 118–121]; however, practitioners should not only coach the plant step when changing direction, but coach a multi-step action taking into account the step(s) prior and step(s) following the plant step. Future investigations that comprehensively examine CODs as a multi-step action (via 3D motion analysis of PFC, plant foot, and following step) are required to improve our understanding of optimal COD techniques.

## Effect of Velocity on COD Biomechanics

Various standardised approach velocities have been administered when exploring COD biomechanics including: 3.0 m·s^−1^ [28, 49, 51, 53], 3.5 m·s^−1^ [122], 3.6–4.4 m·s^−1^ [36, 37], 4.0 m·s^−1^ [47], 4.0–5.0 m·s^−1^ [36, 38, 123], 4.0–5.5 m·s^−1^ [27], 4.5 m·s^−1^ [45], 4.5–5.0 m·s^−1^ [43, 124], 4.5–5.5 m·s^−1^ [27, 90, 125, 126], 5.5–7.0 m·s^−1^ [39, 127] or as fast as possible [25, 30, 42, 56]. However, approach velocity is a critical factor influencing COD biomechanical demands [31–35] (Table 4), which should be acknowledged when interpreting COD biomechanical research and coaching COD technique.

### Faster Approach Velocities Increase Knee Joint Loading during CODs

Vanrenterghem et al. [31] reported increases in knee valgus loading during 45° sidesteps from faster running velocities (4 and 5 m·s^−1^) compared to slower velocities (2 and 3 m·s^−1^). This result is corroborated by previous studies that have reported greater KAMs between faster and slower 60° sidesteps [34], and 135° v cuts with increased approach velocities [35] (Table 4). Additionally, Kristianslund et al. [32] reported approach speed to be a predictor of KAMs during a sports-specific sidestep in handballers. These findings are noteworthy because knee abduction loading can increase strain on the ACL [68, 75, 92, 93] and prospective research has identified KAMs as a predictor of non-contact ACL injury in female adolescent athletes [78]. Furthermore, video analysis investigations have characterised non-contact ACL injuries to occur from CODs with high approach velocities in handball [8] and rugby [10]. Having athletes perform directional changes from slower approach velocities will, indeed, alleviate knee joint loading, but this will compromise COD performance as approach velocity is a determinant of faster COD performance [24, 58]; thus, athletes will be highly unlikely to sacrifice performance at the expense of reduced knee joint loading. Therefore, practitioners and athletes must acknowledge from a technique perspective the ‘performance-injury conflict’ when coaching and performing CODs as fast as possible and ensure athletes have the optimal COD mechanics [32, 37, 38, 44, 45] and physical capacity to tolerate the associative knee joint loading [58, 81–84]. Further research into the most effective training modalities for the optimisation of COD performance and minimising knee joint loading are required.

### Effect on COD Kinetics and Kinematics

While approach velocity is a critical factor on knee joint loading, it also directly influences the kinetic and kinematic profiles demonstrated by athletes (Table 4). Vanrenterghem et al. [31] documented significant increases in peak posterior GRF, ML GRF and concurrent reductions in GCT with increased approach velocities, while knee flexion angles at touchdown were only significantly different at 5 m·s^−1^. Similarly, Dai et al. [33] also demonstrated a significant decrease in GCT and increases in peak posterior GRF, knee extension moment at peak posterior GRF, knee valgus angle and varus moment at peak posterior GRF, knee joint stiffness, and knee flexion angle during 45° sidesteps, when comparing maximum speed versus perceived 60% (Table 4). Kimura and Sakurai [34] also observed significantly shorter GCTs between faster and slower 60° cuts (Table 4). Collectively, these studies highlight the kinetic and kinematic differences during the directional changes with increases in entry velocity, which highlights the difficulty in comparing the results between studies in the literature. Therefore, the injury risk associative studies should be interpreted with respect to the approach velocities used for the COD task, because the velocities examined (≤ 4 m·s^−1^) may not have been high enough to elicit hazardous knee joint loading connected to increased risk of ACL injury [31, 34, 35].

### Role of the PFC

Changing direction is described as a multi-step action [61], with research indicating the step(s) prior to the COD are pivotal in deceleration and initiating effective CODs [34–38, 53, 58–60, 64, 66, 128–133]. Specifically, researchers have shown that the greater braking force characteristics demonstrated in the PFC during COD can alleviate knee joint loading [36–38] and also facilitate faster turning performance [58–60]; however, only two studies have examined the effect of approach velocity on PFC biomechanics. Kimura and Sakurai [34] compared faster and slower 60° sidesteps, reporting greater posterior impulse, shorter GCTs and greater peak external flexion moments in the PFC during the fast condition (Table 4). Similarly, Nedergaard et al. [35] observed greater trunk decelerations at higher approach velocities over the PFC and ipsilateral foot contact during a 135° v cut. Additionally, this was accompanied with greater peak ankle and knee velocities during both of the aforementioned foot contacts, which are suggested to provide an indirect indication of force absorption [67, 134].

Collectively, the results of the aforementioned studies highlight the role of deceleration over the PFC, and the preceding footfalls, to facilitate effective CODs. The PFC not only plays a pivotal role during sharper CODs [25, 34–38, 59, 60, 66], but is fundamental in the execution of directional changes from high approach velocities [34, 35]. This finding is unsurprising as faster entries would require greater braking forces and braking impulse over the PFC and steps prior (change in momentum), to reduce the momentum and entry velocity prior to the COD. Therefore, biomechanists should investigate the PFC when examining COD biomechanics from high approach velocities and sharper CODs for greater insight into the braking force characteristics and mechanisms required to effectively change direction. Furthermore, practitioners are encouraged to coach a deceleration strategy that emphasises braking forces over several gait cycles, in particular the PFC, when coaching sharp CODs or sharp CODs from fast approach velocities. Previous researchers have shown technique changes and reduced knee joint loading in cutting [44] and turning [135] from a 6-week (two sessions per week) COD technique modification intervention. Further research is needed to confirm whether longitudinally coaching PFC-dominant deceleration strategies are effective for improving COD performance and reducing knee joint loading.

## Effect of Velocity and Angle on COD Performance

Straight line sprint speed is suggested to be a determinant of COD performance as it would be advantageous to enter and exit the COD as fast as possible [119, 136]. However, there is a paucity of studies that have examined an athlete’s horizontal velocity before, during and after a COD [24, 58]. Hader et al. [24] assessed COM velocity during a 45° and 90° cut from a 10-m approach using laser speed guns, reporting the minimum speed during the COD was largely associated with cutting performance for both tasks, and peak acceleration and peak speed also contributed to faster performance. These findings highlight the importance of maintaining and minimising the decline in velocity prior to and during a 45° and 90° cut for faster performance. Thus, coaching strategies that encourage the maintenance of velocity by limiting preliminary deceleration may be warranted for faster cutting performance. However, it should be noted that some deceleration (reductions in velocity) will be present when changing direction [24, 25], in particular for sharper 90° cuts (Fig. 1), but the ability to tolerate the greater velocities may facilitate faster performance.

Examining a sharper 180° COD in female soccer players, Jones et al. [58] inspected the horizontal velocity of COM from the approach of the PFC to the exit of the FFC, using the methods described by Vanrenterghem et al. [137]. Faster performance was inversely associated (*r* = −0.484, *r*^*2*^ = 23%) with greater approach velocities (i.e. horizontal model COM velocity at the start of the PFC). Notably, eccentrically stronger (knee extensor peak torque) soccer players demonstrated faster COD performance (*d* = −2.09), greater approach velocities (*d* = 1.27), and greater reductions in velocity during the PFC (*d* = −0.94) in comparison to weaker, while greater peak and average horizontal GRFs over the PFC were also displayed by stronger athletes (*d* = 1.00–1.23). These findings suggest there may be an interaction between strength and speed in the facilitation of faster COD performance. Based on the results that eccentrically stronger athletes demonstrated better deceleration abilities, approached with greater velocities, and produced greater change in velocities and braking forces, the authors introduced a concept of ‘self-regulation’ regarding approach velocity (i.e. ‘a player approaches faster based on the deceleration load they know/feel they can tolerate’). Nonetheless, the ability to approach a 180° turn quickly and reduce the velocity over the PFC into the FFC is integral for faster performance, while the ability to decelerate efficiently is underpinned by eccentric strength capacity.

The majority of investigations have examined the influence of angle on injury risk factors during CODs [19, 22, 25, 27, 29]; however, a paucity of research exists examining the influence of angle on COD performance, whereby the optimal techniques for faster performance will most likely be angle dependent [21, 24, 26, 30]. Rouissi et al. [138] examined completion times and COD deficit during a range of 10-m COD tasks (5-m entry and exit) of different angles (45°, 90°, 135° and 180°), noting a trend in increasing completion times and COD deficits in male soccer players as COD angle increased. Likewise, Schreurs et al. [22] reported significant increases in completion time as COD angle (45°, 90°, 135° and 180°) increased in male and female athletes. These findings are unsurprising because as the COD angle increases, greater reductions in velocity (change in momentum) are required [23–25], thus increasing the demands for preliminary deceleration, which typically occur over greater distances [23–25]. In addition, GCTs also increase with sharper CODs (Table 2); therefore, the combination of deceleration and longer GCTs most likely explain the longer completion times associated with sharper CODs.

The technique required for faster COD performance is also angle dependent [21, 24, 26, 30]. For example, Havens and Sigward [30] found that the determinants for 45° and 90° cutting performance differed between tasks. Faster 45° completion times were associated with greater hip sagittal power, hip extensor moments and greater ML COM-COP distances. Conversely, faster 90° cut performance was associated with greater hip frontal power and ML GRI, indicating a greater reliance on frontal plane biomechanics for 90° cutting, in contrast to sagittal plane determinants for 45° cutting. In addition, Hader et al. [24] identified that the minimum speed during the COD was a predictor of faster cutting performance. Therefore, when maintaining velocity is essential such as running around the bases in baseball/softball or possessing greater momentum in collision sports such as rugby and American football is desired, a rounded or shallow COD is recommended due to the shorter GCTs and minimal reductions in velocity [21, 24, 52]. Conversely, when the aim is to execute sharper CODs, in particular ≥ 60° to evade an opponent or turn in response to a ball or opponent, substantial deceleration over several gait cycles prior to the plant foot contact will undoubtedly be required [25, 34–38, 58–61, 66].

## Changing Direction: An Angle-Velocity Trade-Off During Cutting

In multidirectional sport it would be advantageous to perform sharp cuts from high approach velocities with minimal reductions in velocity [5, 24, 132]; however, an angle-velocity trade-off exists when performing cutting manoeuvres [19, 31, 55] (Table 3). Vanrenterghem et al. [31] reported a reduction in task execution (executed cutting angle) with increased running velocities during 45° sidesteps (Table 3). Similarly, Suzuki et al. [55] found greater cutting angles with a sidestep technique compared to a crossover cut during an intended 90° COD, but a greater reduction in velocity was also demonstrated (Table 3). Furthermore, Schot et al. [19] stated that in pilot work, in order to achieve a 90° cut as fast as possible, subjects would often require three to five steps to perform the directional change, but this resulted in a rounded COD. Therefore, it is evident a trade-off between angle and velocity exists when changing direction, whereby executing CODs from fast approach velocities reduces task execution (executed cutting angle), and vice versa. This finding has large implications for the deceleration requirements for COD, as the deceleration strategy to execute the COD effectively is governed by the angle and velocity at which the COD is performed.

Maintaining velocity and greater approach velocities are identified as determinants of COD performance [24, 58] and, therefore, may be encouraged when coaching cutting technique. However, faster approach velocities may compromise the executed cutting angle and also increase knee joint loading, which creates a conflict for practitioners and athletes. As such, practitioners must identify the aim of the COD attribute they are aiming to develop (i.e. velocity maintenance, COD angle execution, or balance of the two factors), and consider the context and specific movement demands of the sport. For example, in sports where the maintenance of velocity is essential when performing COD such as running around the bases in softball/baseball, or a subtle COD to maintain momentum in collision sports such as rugby and American football, a rounded or shallow COD, with high approach velocities, thus momentum and shorter GCTs may be warranted [21, 24, 52]. Where a subtle COD is necessary, a crossover cut is recommended due to greater velocity maintenance, and this technique results in shorter GCTs [55, 102, 117] (Fig. 1). Conversely, scenarios where sharper cuts are necessary to evade (deceive) and create larger separation distances from opponents, a slower approach or reduction in approach velocity may be necessary (potentially over several gait cycles), and a sidestep strategy to facilitate an effective sharp COD is recommended [55, 102, 117] (Fig. 1).

## Implications of Change of Direction Angle for Training Design and Exercise Selection

The determinants of COD performance are multifaceted [2, 4, 83, 136]; however, physical attributes such as rate of force development and reactive strength (fast or slow stretch-shortening cycle abilities) are fundamental qualities underpinning COD performance [2, 4, 83, 136], while the large levels of relative lower body loading associated with COD [28, 32, 37, 44, 45] actions must also be acknowledged. As such, training modalities that enhance COD performance and an athlete’s robustness to tolerate the loading associated with CODs are of great interest to practitioners.

Table 2 outlines training and exercise selection recommendations for athletes who participate in multidirectional sport, and we specifically introduce a novel concept of selecting training exercises in accordance to COD angle due its subsequent effect on GCTs. Lower limb plyometric training is an effective training modality for enhancing COD performance [139–141], due to the similarities in GCT and the involvement of an eccentric-concentric coupling action [111, 142]. Specifically, lower limb plyometric exercises provide a stimulus resulting in high power outputs, high ankle flexor moments in short GCTs [143], increased force output and stretch shortening cycle (SSC) efficiency [139], all of which are important components for faster COD performance. Furthermore, plyometric and jump-landing training with appropriate feedback can also enhance lower limb control, reduce knee valgus and reduce impact forces and torques [84, 144–147], thus reducing the potential risk of injury. Table 2 provides exercise selection recommendations dependent on COD angle: for example, in light of the GCTs reported for shallow CODs (≤ 60°) (Table 2), fast SSC (fast reactive strength < 0.25 s) exercises are recommended [148]. Conversely, for sharper CODs (≥ 135°), slow SSC actions and ballistic exercises (slow reactive strength > 0.25 s) are recommended, whereas a combination of fast and slow SSC exercises are recommended for 90° cuts, due to bordering fast and slow SSC classification (~0.25–0.35 s) [148].

Cutting is a unilateral, multiplanar movement, and dependent on the ability to generate ML propulsive force and impulse [25, 30, 38], hip frontal plane power [30] and ankle power [40]. Therefore, plyometric exercises should not only be performed in the sagittal plane emphasizing vertical displacement, but also performed in several directions [139] in the frontal and transverse plane emphasising horizontal displacement [111]. In particular, unilateral plyometrics (training recommendations in Table 2) should be incorporated into the strength and conditioning program due to the similarity in the orientation of force application and push-off action to cutting [136]. However, practitioners should be aware that landings in the frontal plane may have higher task complexity and have a higher risk of knee injury in comparison to forward and diagonal landings [149].

Athletes should ideally possess a solid foundation of strength (one repetition maximum back squat ≥ 1.5 × body mass) before performing complex and higher intensity plyometrics [81, 101, 118], while eccentric strength capacity is also fundamental for successful COD performance and tolerating the large joint loading [41, 58, 81, 98, 105–109]. Shorter GCTs have been identified as determinants of faster COD performance [40, 42, 59, 112]; thus, the aims of the aforementioned training recommendations are to reduce braking and propulsive force duration (total duration), but simultaneously increase braking and propulsive forces, resulting in a tall and thin impulse, in contrast to a short and wide impulse. In summary, practitioners should understand the implications of COD angle on GCT and should therefore select exercises according to COD angle and task demands. Moreover, practitioners are encouraged to implement a holistic multi-component strength and conditioning programme that integrates strength, plyometric, trunk (core) and COD technique training for the enhancement of COD performance and injury risk reduction [44, 81, 84, 87, 150].

## Conclusions

The biomechanical demands of CODs are ‘angle’ and ‘velocity dependent’ and are both critical factors that influence the technical execution of the COD, deceleration and reacceleration requirements, knee joint loading, and lower limb muscle activity. Thus, these two factors regulate the progression and regression in COD intensity. Specifically, faster and sharper CODs increase knee joint loading, but are also required for successful performance creating a ‘performance-injury conflict’ from a technique perspective; however, this conflict can be mediated by an athlete’s physical capacity (i.e. ability to rapidly produce force and neuromuscular control) and performing the COD with optimal mechanics. Furthermore, an ‘angle-velocity trade-off’ exists during CODs, whereby faster approaches compromise the execution of the intended COD; this is influenced by an athlete’s physical capacity. Therefore, practitioners and researchers should acknowledge and understand the implications of angle and velocity on COD biomechanics when: (1) interpreting biomechanical research; (2) coaching COD technique; (3) designing and prescribing COD training and injury reduction programs; (4) conditioning athletes to tolerate the physical demands of directional changes; (5) screening COD technique; and (6) progressing and regressing COD intensity, specifically when working with novice or previously injured athletes who are rehabilitating from an injury.
